# Glucose and glutamine handling in the Sertoli cells of transgenic rats overexpressing regucalcin: plasticity towards lactate production

**DOI:** 10.1038/s41598-018-28668-4

**Published:** 2018-07-09

**Authors:** Inês Mateus, Mariana Feijó, Luís M. Espínola, Cátia V. Vaz, Sara Correia, Sílvia Socorro

**Affiliations:** CICS-UBI - Health Sciences Research Centre, University of Beira Interior, Covilhã, Portugal

## Abstract

Sertoli cells (SCs) possess the unparalleled ability to provide the germ line with growth factors and nutrients. Although SCs can oxidize amino acids, e.g., glutamine, they mostly metabolize glucose, producing high amounts of lactate, the germ cells preferential substrate. Regucalcin (RGN) is a calcium-binding protein that has been indicated as a regulator of cell metabolism. In this study, we investigated glucose and glutamine handling in the SCs of transgenic rats overexpressing RGN (Tg-RGN) comparatively with wild-type (Wt) littermates. Primary SCs isolated from adult Tg-RGN animals and maintained in culture for 24 hours, produced and exported more lactate, despite consuming less glucose. These observations were underpinned by increased expression of alanine transaminase, and augmented glutamine consumption, suggesting that alternative routes are contributing to the enhanced lactate production in the SCs of Tg-RGN rats. Moreover, lactate seems to be used by germ cells, with diminished apoptosis being detected in the seminiferous tubules of Tg-RGN animals cultured *ex vivo*. The obtained results showed a distinct metabolism in the SCs of Wt and Tg-RGN rats widening the roles assigned to RGN in spermatogenesis. These findings also highlighted the plasticity of SCs metabolism, a feature that would be exploited in the context of male infertility.

## Introduction

The seminiferous epithelium is fundamentally constituted by two cellular components: the germinal lineage and the somatic Sertoli cells (SCs), which play a determinant role in the complex and strictly regulated process of germ cell development^1–3^. Indeed, a successful spermatogenesis relies on a multiplicity of SCs functions that range from physical support and immunoprotection to the supply of growth factors and nutrients^1,4,5^.

Glucose is the universal energy substrate nearly used by all cells, but despite developing germ cells express all enzymes of the glycolytic pathway, they are dependent on the nutrients provided by the secretory activity of SCs^6,7^. Lactate obtained from the extracellular medium has been indicated as the main energy substrate for germ cells^8,9^, which have their energetic needs ensured by the ability of SCs to drive the metabolism of glucose to the production and export of lactate^10,11^. Glucose is uptaken by SCs via specific high-affinity glucose transporters (GLUTs) localized at the plasma membrane^12^, and once inside the cells it goes through the sequential reactions of glycolysis. The end-product of glycolysis pyruvate, instead entering the citric acid cycle (TCA) cycle is preferentially metabolized to lactate by lactate dehydrogenase (LDH)^9–11^. Lactate produced by SCs is then exported onto the extracellular space through the activity of membrane monocarboxylate transporters (MCTs) and made available to the developing germ cells^13^. Also, amino acids can be used as an energy source by SCs. Among others, SCs have the ability to uptake external glutamine via a specific membrane transporter, the ASC amino-acid transporter 2 (ASCT2)^14^, and the oxidation of glutamine seems to provide most of the energy required by these cells^15^.

Over the past few years, SCs metabolism has emerged as an important modulator of male fertility, since the stimulation of the glycolytic pathway boosts the development of both SCs and germ cells^9,16–18^. Also, it has been shown that, besides being an energy substrate, the lactate produced by the SCs has anti-apoptotic effects over the germ line^8,9^. Although SCs’ metabolism and lactate production have been shown to be modulated by several regulators, such as, growth factors^13,19^, cytokines^13,20^ and sex steroid hormones^21^, the regulation of SCs metabolism still is a subject that needs further elucidation.

Regucalcin (RGN) is a calcium(Ca^2+^)-binding protein first described in 1978^22^ that has been indicated as a regulator of cell proliferation and apoptosis^23–27^. RGN plays a regulatory role in Ca^2+^-dependent and -independent signalling processes in many types of cells^27–32^ and is expressed in numerous tissues. Previous findings from our research group confirmed that RGN is expressed both in SCs and germ cells and suggested that it may play a relevant role in spermatogenesis^32,33^. More recently, it was shown that RGN overexpression suppresses chemical-^25^ or radiation-induced^34^ apoptosis, and oxidative stress^35^ in the testis, which suggests a protective role of this protein for germ cells. Although several approaches also have demonstrated that RGN modulates glucose handling by regulating the expression of several transporters and glycolytic enzymes^26,36,37^, RGN’s influence in the glycolytic metabolism, and glutaminolysis, of testicular cells is totally unknown. The present study aims to characterize glucose and glutamine metabolism in the SCs of transgenic rats overexpressing regucalcin (Tg-RGN) comparatively with their wild-type (Wt) littermates, widening the knowledge of RGN actions in the testis and further detailing the regulation of SCs metabolism. The deep understanding of the mechanisms that control the metabolic function of these “nurse cells” is pivotal for the development of infertility treatments targeting metabolism.

## Results

### Altered glucose consumption and lactate production in the SCs of Tg-RGN rats

**Figure 1:**
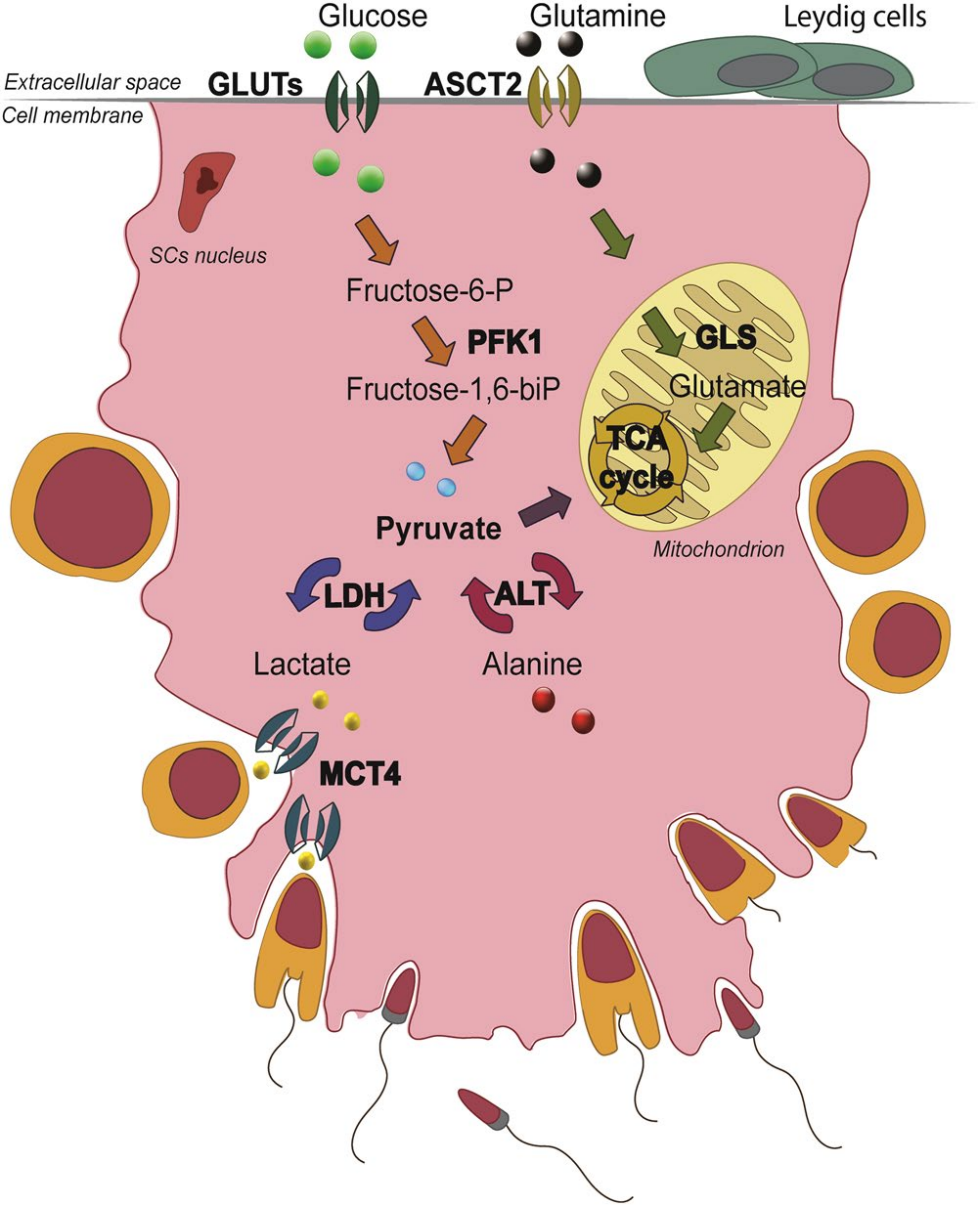
Schematic representation of the glucose and glutamine metabolizing pathways in Sertoli cells (SCs). The exogenous glucose is uptaken by SCs via specific glucose transporters (GLUTs), namely, the GLUT1, GLUT2 and GLUT3, being then converted to pyruvate by glycolysis (orange arrows) through the sequential action of several enzymatic players. This includes the phosphofructokinase-1 (PFK1) that catalyses a rate limiting step of glycolysis converting fructose 6-phosphate to fructose 1,6-bisphosphate. Pyruvate can either be directed into the mitochondrion to regenerate acetyl-CoA, or can be converted into lactate by lactate dehydrogenase (LDH). Pyruvate can also be obtained via alanine by the reversible reaction catalysed by alanine transaminase (ALT), respectively. SCs seem preferentially use pyruvate to produce lactate that is exported onto the extracellular space via monocarboxylate transporters (MCTs), specifically by the MCT4. Among other substrates, the SCs also present the ability to metabolize glutamine (green arrows). This amino acid enters the cell via specific glutamine transporters (ASCT2) and is directed to the citric acid cycle (TCA) cycle for ATP generation.

Sertoli cells’ glucose metabolism (Fig. 1) is essential for a successful spermatogenesis, since developing germ cells consume lactate produced by the SCs as their main energy source^6^. In order to assess if RGN modulates the glycolytic metabolism of SCs, we started by measuring the content of glucose and lactate in primary SCs of Wt and Tg-RGN animals, and in the respective culture medium at 24 hours of culture.

**Figure 2:**
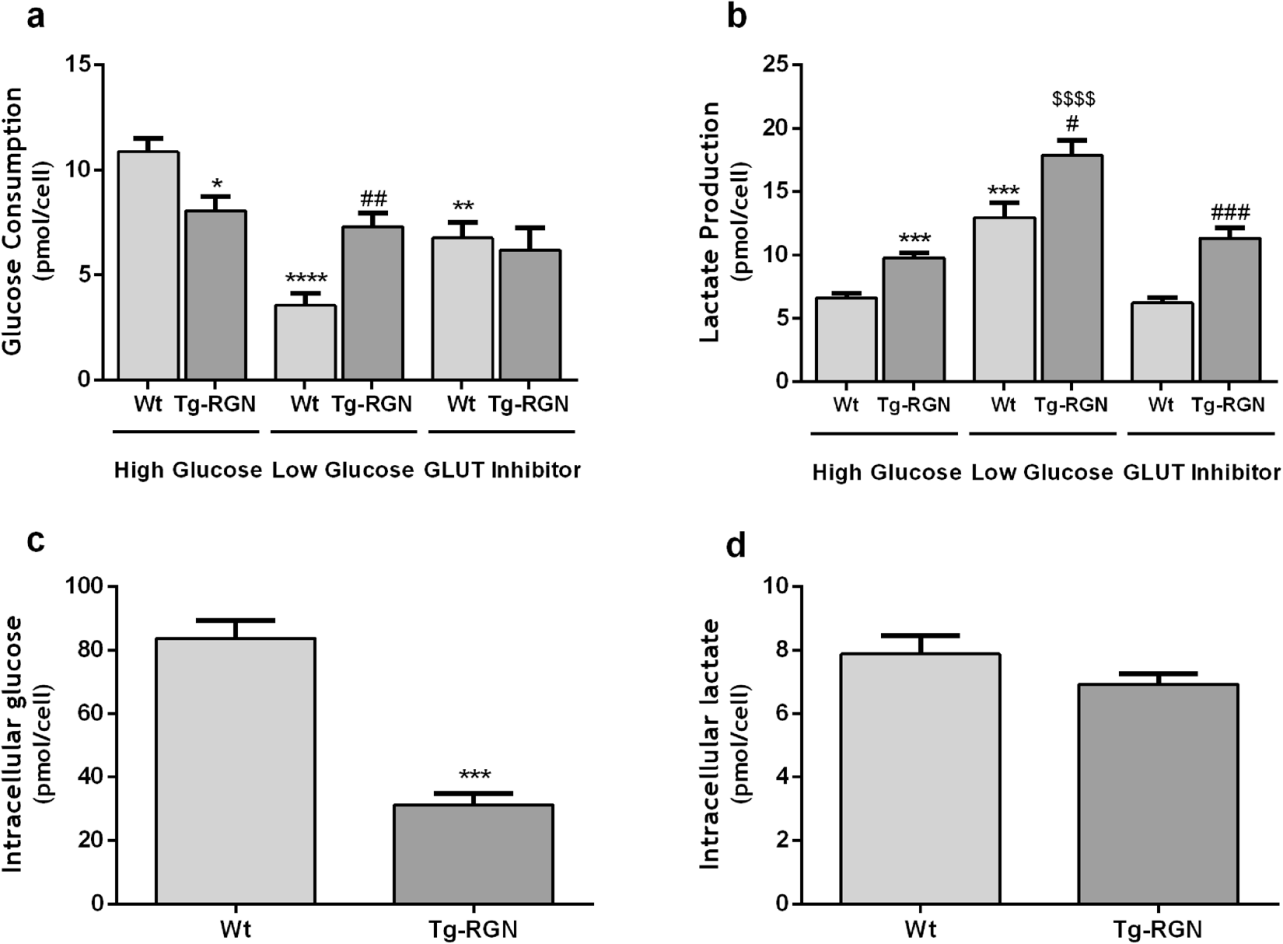
Glucose consumption (**a**) and lactate production (**b**) in the primary SCs of Wt and Tg-RGN rats cultured for 24 hours in the presence of high (17 mM) or low (8 mM) glucose concentrations, and GLUT1/3 inhibitor (in high glucose); intracellular concentration of glucose (**c**) and lactate (**d**) in the SCs of Wt and Tg-RGN rats cultured for 24 hours. Data are presented as mean ± S.E.M. (n ≥ 4 in each group). *P < *0.05*, **P < *0.01*, ***P < *0.001, and* ****P < *0.0001* when compared with Wt group in the presence of high glucose; ^*#*^*P* < *0.05*, ^*##*^*P* < *0.01, and* ^*###*^*P* < *0.001* when compared with Wt group in the presence of low glucose; ^*$$$$*^*P* < *0.0001* when compared with the Tg-RGN group in the presence of high glucose.

Under conditions of high glucose availability (17 mM), glucose consumption (Fig. 2a) was significantly lower in the SCs of Tg-RGN rats when compared with their Wt littermates (8,05 ± 0,69 vs. 10,85 ± 0,65 pmol/cell, P < 0.05). The SCs of Wt animals displayed diminished glucose consumption in response to lower glucose concentrations (8 mM) in the culture medium (3,56 ± 0,59 vs. 10,85 ± 0,65 pmol/cell, P < 0.0001, Fig. 2a), an effect not seen in the Tg-RGN animals. Therefore, gene expression analysis of glycolytic metabolism-related proteins presented below was performed for SCs cultured with 17 mM. Moreover, glucose consumption by the SCs of Tg-RGN also was not affected in the presence of GLUT1/3 inhibitor (Fig. 2a). Contrastingly, GLUT1/3 inhibitor significantly diminished the glucose consumption by the SCs of Wt (6,75 ± 0,76 vs. 10,85 ± 0,65 pmol/cell, P < 0.01, Fig. 2a).

Considering lactate production (Fig. 2b), it was increased by approximately 48% (9,77 ± 0,41 vs. 6,62 ± 0,35 pmol/cell, P < 0.001) and 38% (17,85 ± 1,23 vs. 12,95 ± 1,16 pmol/cell, P < 0.05) in the SCs of Tg-RGN animals comparatively with control animals under high and low glucose concentrations, respectively. Lactate production was not affected by the presence of GLUT1/3 inhibitor, both in Tg-RGN and Wt animals (Fig. 2b). However, both Wt and Tg-RGN rats SCs cultured in reduced glucose availability (8 mM) displayed significantly increased lactate production (Fig. 2b, 12,95 ± 1,16 vs. 6,62 ± 0,35 pmol/cell, P < 0.001 and 17,85 ± 1,23 vs. 9,77 ± 0,42 pmol/cell, P < 0.0001, respectively).

Also, the intracellular content of glucose (Fig. 2c) showed to be significantly decreased in the SCs of Tg-RGN when compared with Wt animals (83,82 ± 5,62 vs. 31,27 ± 3,55 pmol/cell, P < 0.001). No changes were observed in the intracellular content of lactate (Fig. 2d) between both experimental groups.

**Figure 3:**
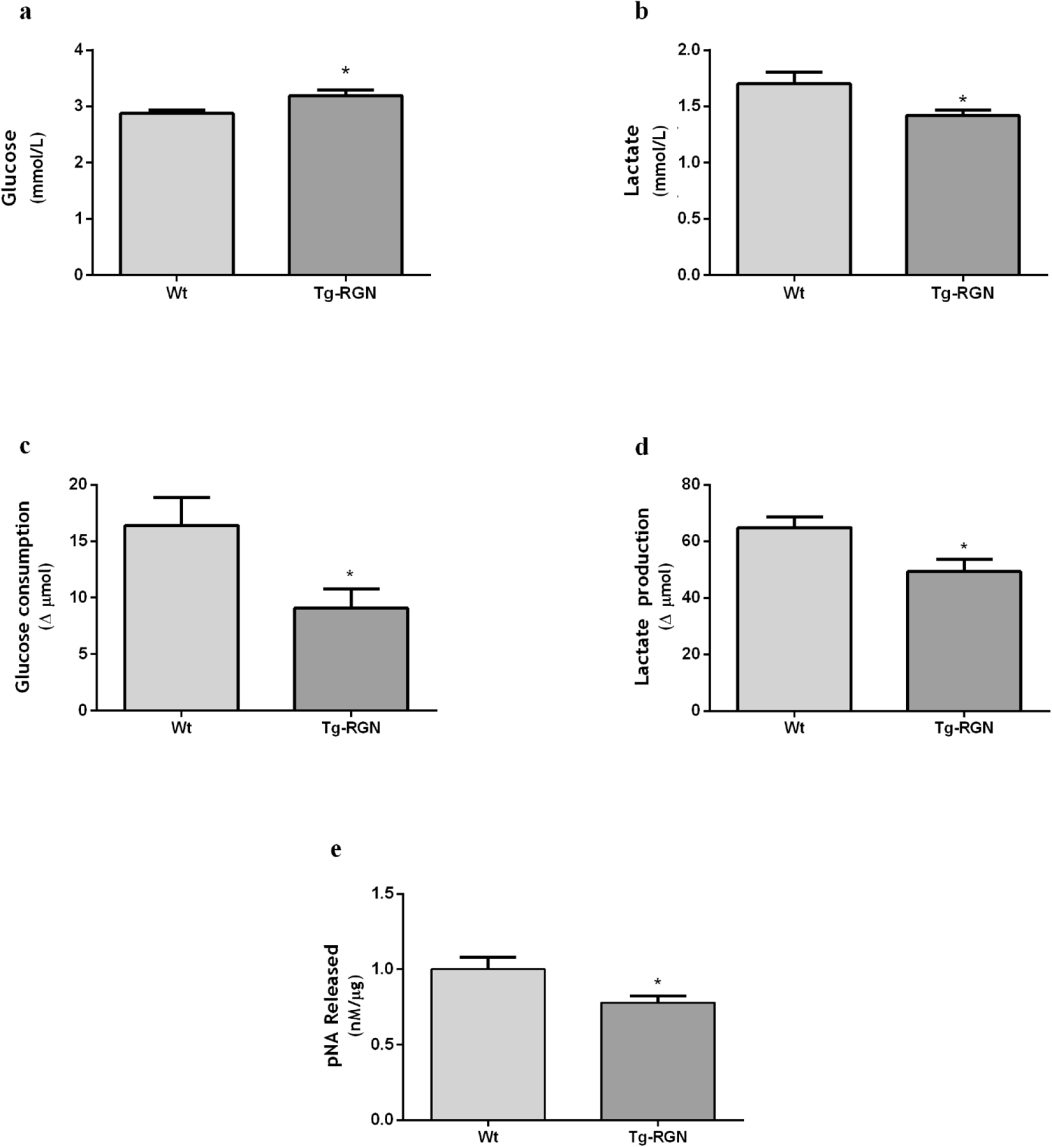
Glucose (**a**) and lactate (**b**) content in the SeT fluid of Wt and Tg-RGN rats, and glucose consumption (**c**) and lactate production (**d**) in SeT cultured with 17 mM glucose for 48 hours. (**e**) Caspase-3 activity in the SeT of Wt and Tg-RGN determined by a colorimetric assay measuring the release of pNA product after cleavage of the Ac-DEVD-pNA substrate (nM/μg) and represented as variation to control. Data are presented as mean ± S.E.M. (n ≥ 4 in each group). *P < 0.05.

The activity of SCs is known to establish the composition of the seminiferous tubules (SeT) fluid and provide the energetic substrates for developing germ cells^6,38^. Therefore, the content of glucose and lactate in the SeT fluid of Tg-RGN and Wt rats was determined also (Fig. 3). The SeT fluid of Tg-RGN animals showed increased glucose (3,19 ± 0,10 vs 2,87 ± 0,06, P < 0.05, Fig. 3a) and diminished lactate concentration (1,42 ± 0,05 vs 1,70 ± 0,10, P < 0.05, Fig. 3b) when compared to Wt animals. To have an indication of the physiological relevance of lactate production by SCs, as well as, the distinct content of metabolites in the SeT fluid of Tg-RGN animals, *ex vivo* cultures of SeT were performed. Glucose consumption and lactate production were determined, and apoptosis rate was evaluated (Fig. 3). In the SeT of Tg-RGN, both glucose consumption (Fig. 3c) and lactate production (Fig. 3d) were significantly lower comparatively with the respective Wt control groups (9,08 ± 1,70 vs 16,39 ± 2,47 and 49,26 ± 4,29 vs 64,90 ± 3,62, respectively, P < 0.05). These metabolic alterations in the SeT of Tg-RGN rats were underpinned by diminished apoptotic rates, as indicated by the lower activity of the executioner of apoptosis, caspase-3 (22% reduction compared with the control, P < 0.05, Fig. 3e).

### SCs of Tg-RGN rats showed decreased expression of GLUT2 and increased levels of PFK1, ALT and MCT4

**Figure 4:**
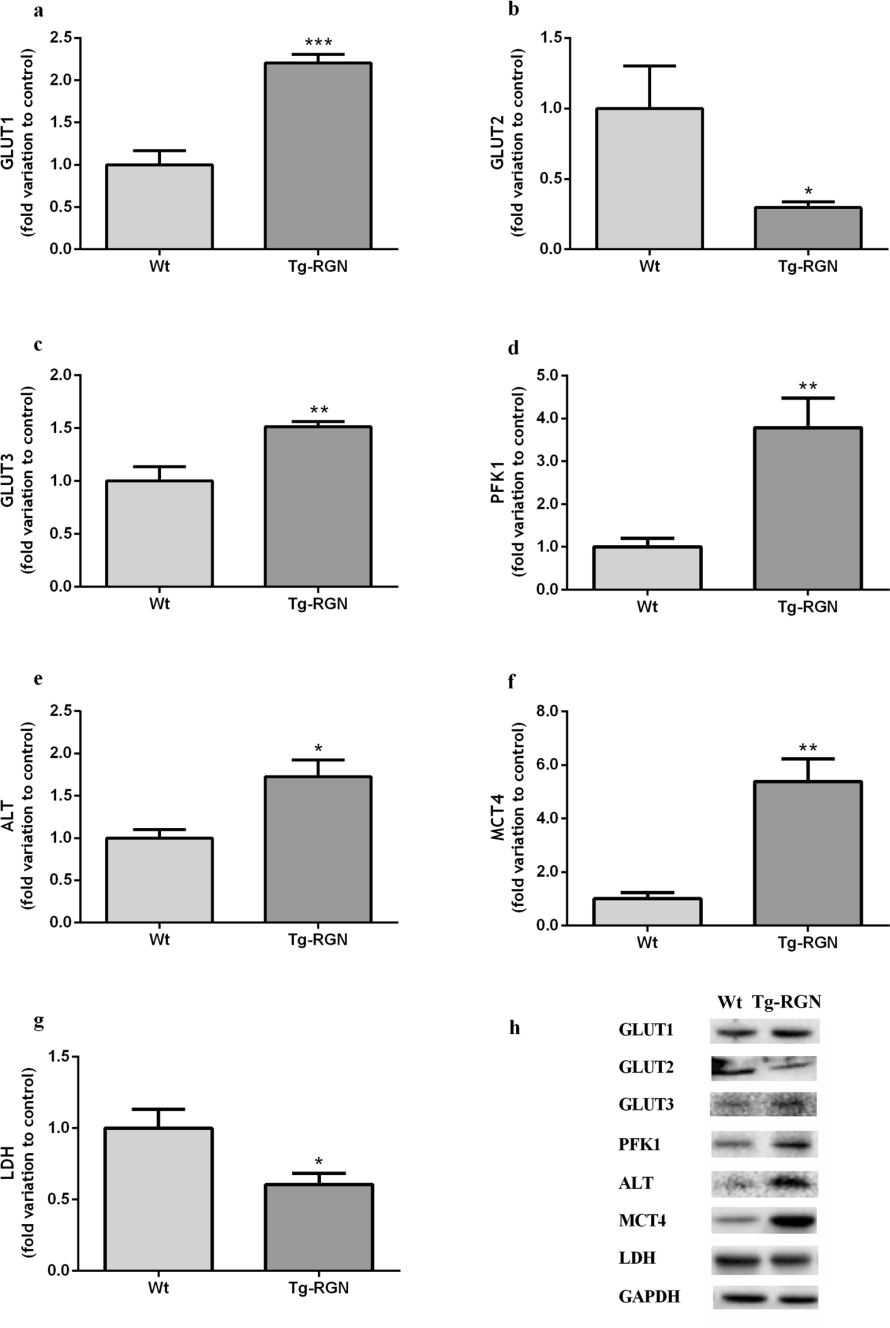
Expression of metabolism-related proteins, GLUT1 (**a**), GLUT2 (**b**), GLUT3 (**c**), PFK1 (**d**), ALT (**e**), MCT4 (**f**), and LDH (**g**) in primary SCs of Wt and Tg-RGN rats cultured with 17 mM glucose for 24 hours. Data are presented as mean ± S.E.M. after normalization with GAPDH (n ≥ 4 in each group). Results are expressed as fold-variation relatively to Wt animals. *P < 0.05, **P < 0.01, ***P < 0.001. Representative immunoblots are shown in panel (h).

The possible role of RGN in the regulation of glycolytic metabolism (Fig. 1) was evaluated through the analysis of GLUT1, GLUT2, GLUT3, phosphofructokinase-1 (PFK1), MCT4 and alanine transaminase (ALT) expression levels (Fig. 4) in the SCs of Tg-RGN and Wt.

GLUTs isoforms are characterized by displaying substrate specificity, proper kinetic characteristics and tissue-specific expression pattern^39^. The protein levels of GLUT1 and GLUT3, those that have been mainly implicated in SCs metabolism^12,21,40^, were significantly increased in the SCs of Tg-RGN rats when compared to the control group (2,21 ± 0,10 fold variation, P < 0.001, Fig. 4a, and 1,51 ± 0,05 fold variation, P < 0.01, Fig. 4c, respectively). In opposition, GLUT2 expression was reduced by ~70% in the SCs of Tg-RGN rats (P < 0.05, Fig. 4b).

PFK1 is an extremely important regulatory enzyme long established to determine the flux through the glycolytic pathway (Fig. 1). The expression levels of this enzyme were significantly higher in the SCs of Tg-RGN animals when compared to control (3,78 ± 0,70 fold variation, P < 0.01, Fig. 4d).

The main product of glycolysis pyruvate can also be generated by the activity of ALT, an enzyme responsible for the reversible catalysis of alanine into pyruvate. Due to this ability, ALT plays a key role in the intermediary metabolism of glucose and amino acids (Fig. 1)^41,42^. The SCs of Tg-RGN animals presented higher expression levels of ALT when compared to the SCs of their Wt littermates (1,73 ± 0,20 fold variation, P < 0.05, Fig. 4e).

The MCT4 is the MCT family member required for lactate export in highly glycolytic cells, which presence in SCs was previously confirmed^21,43,44^. The SCs of Tg-RGN rats showed a pronounced increase in MCT4 expression levels when compared to the control group (5,38 ± 0,85 fold variation, P < 0.01, Fig. 4f).

### The protein expression and enzymatic activity of LDH were decreased in the SCs of Tg-RGN rats

**Figure 5:**
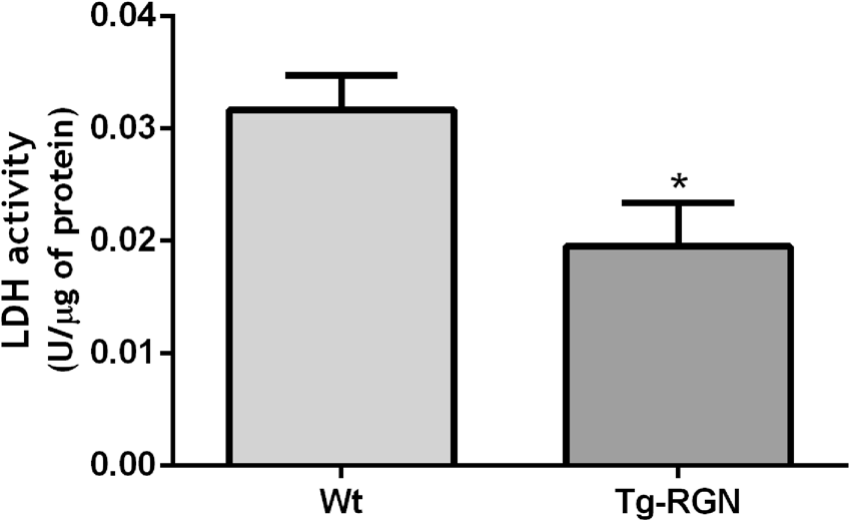
Enzymatic activity of LDH in primary SCs of Wt and Tg-RGN rats after culture for 24 hours in 17 mM glucose. Data are presented as mean ± S.E.M. (n ≥ 4 in each group). *P < 0.05.

The expression levels of LDH (Fig. 4g), the enzyme responsible for the reversible conversion of pyruvate into lactate^45^, were significantly lower in the SCs of Tg-RGN cultured for 24 hours (0,61 ± 0,08 fold variation to control, P < 0.05). Accordingly, a significant decrease in the enzymatic activity of LDH (Fig. 5) was observed in the SCs of Tg-RGN when compared to the Wt experimental group (0,032 ± 0,003 vs. 0,019 ± 0,003 U/μg of protein, P < 0.05).

### Enhanced glutamine consumption and augmented protein expression of ASCT2 and GLS in the SCs of Tg-RGN animals

**Figure 6:**
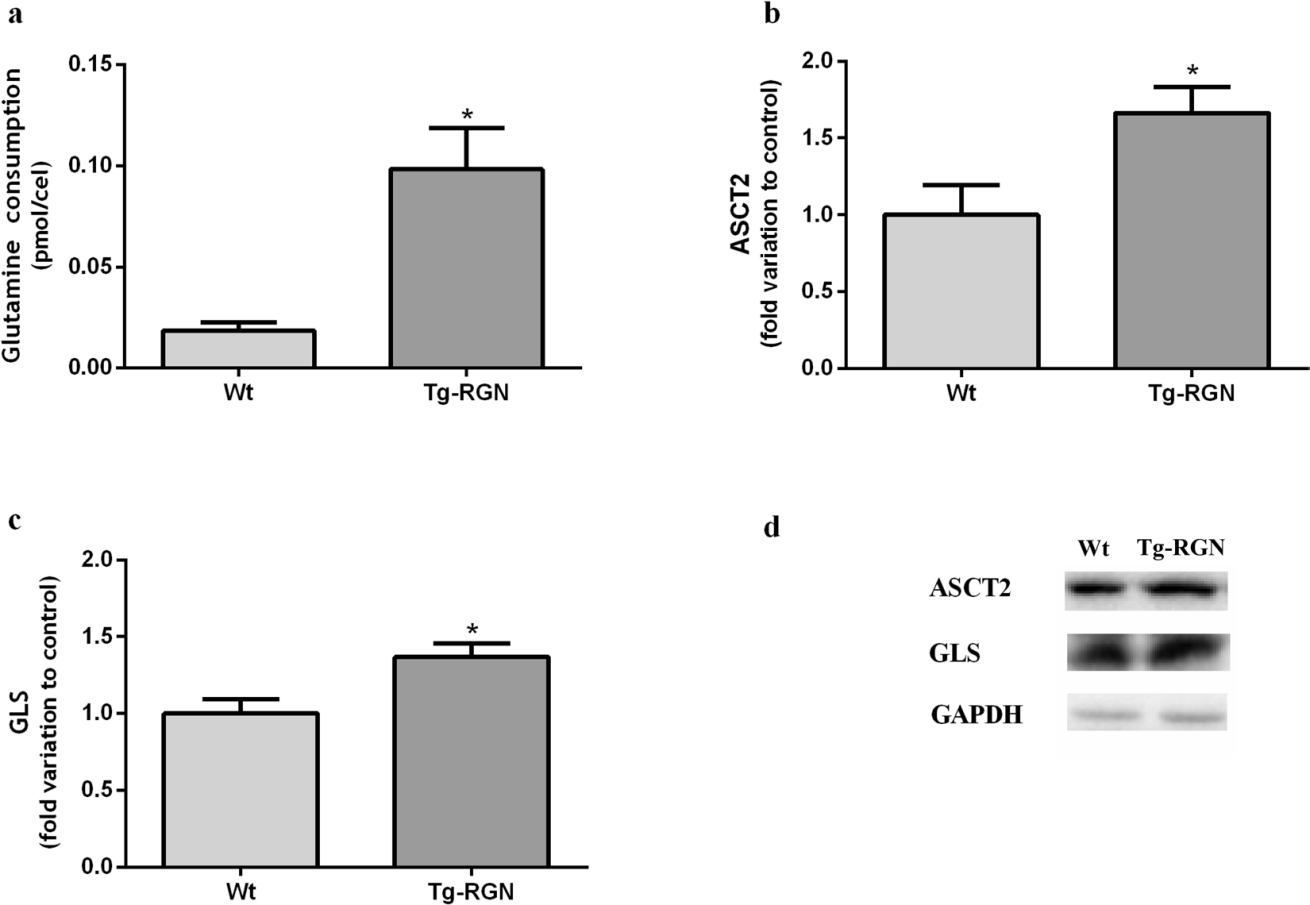
Glutamine consumption (**a**) and expression of glutaminolysis-related proteins, ASCT2 (**b**) and GLS (**c**) in primary SCs of Wt and Tg-RGN rats cultured with 17 mM glucose for 24 hours. Data are presented as mean ± S.E.M. after normalization with GAPDH (n ≥ 4 in each group). Results are expressed as fold-variation relatively to Wt animals. *P < 0.05. Representative immunoblots are shown in panel (d).

The oxidation of glutamine is other important metabolic pathway used by SCs. We determined the glutamine consumption by SCs of Tg-RGN animals comparatively with the control group at 24 hours of culture, which was significantly augmented (0,10 ± 0,02 vs 0,02 ± 0,004 pmol/cell in the control, P < 0.05, Fig. 6a).

The expression levels of glutamine transporter ASCT2 (Fig. 6b) were significantly higher in the SCs of Tg-RGN after 24 hours of culture (1,66 ± 0,17 fold variation to control, P < 0.05). Glutaminase (GLS) is a mitochondrial enzyme that catalyses a crucial step of glutaminolysis: the conversion of glutamine into glutamate (Fig. 1)^46^. The expression levels of GLS (Fig. 6c) were significantly higher in the SCs of Tg-RGN cultured for 24 hours (1,37 ± 0,1 fold variation to control, P < 0.05).

**Figure 7:**
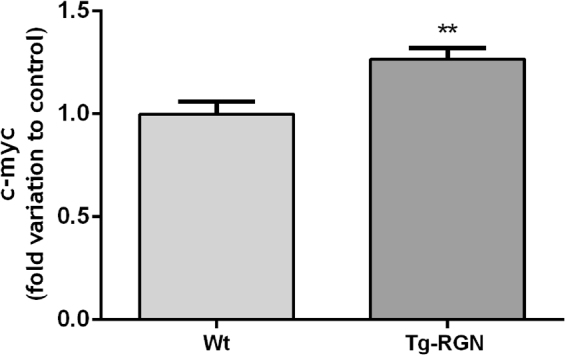
Transcript levels of c-myc in primary SCs of Wt and Tg-RGN rats cultured with 17 mM glucose for 24 hours. Data are represented as mean ± S.E.M. after normalization with β-actin and GAPDH (n ≥ 5 in each group). Results are expressed as fold-variation relatively to Wt animals (**P < 0.01).

The transcription factor c-myc has been implicated in the stimulation of glutaminolysis^47^ and also has been shown to be modulated by RGN overexpression^48^. Therefore, we decided to analyse c-myc expression (Fig. 7) in the SCs of Tg-RGN comparatively with Wt, which was significantly increased (1,3 fold variation to control, P < 0.01).

## Discussion

Although RGN’s actions have been associated with protection of spermatogenesis and the modulation of cellular metabolic processes^24–26,35,49,50^, its relationship with the glycolytic metabolism of SCs remains completely unexplored. In the present study, we started by investigating glucose metabolism in SCs under overexpression of RGN comparatively with that of Wt controls. It was found that the SCs of Tg-RGN animals consumed less glucose, and that this behaviour was not affected by the lower availability of glucose (Fig. 2a). Contrastingly, SCs of Wt animals cultured in low glucose showed significantly reduced consumption of this metabolite (Fig. 2a), which do not follow previous findings^51^ and do not has a definitive explanation.

Glucose consumption firstly depends on the glucose uptake from the extracellular space, a process mediated by the GLUTs present at the cell membrane (Fig. 1). At least four GLUTs isoforms have been identified in the SCs (GLUT1, GLUT2, GLUT3 and GLUT8)^40,52^ though only GLUT1 and GLUT3 have been reported as playing a crucial role in glucose incorporation^12^. We have observed that both GLUT1 and GLUT3 expression levels were elevated in the SCs of Tg-RGN rats (Fig. 4a and c, respectively) which, at first sight, would appear not consistent with the diminished glucose consumption observed. As mentioned above, GLUT1 and GLUT3 were the GLUTs that have been associated with the glucose transport in rat SCs being expressed throughout pubertal development, and with GLUT1 also indicated as a target of the hormonal regulation by FSH^12^. However, we also have found that GLUT2 expression was strongly diminished in the SCs of Tg-RGN animals in the presence of 17 mM glucose (Fig. 4b), and in agreement with the diminished glucose consumption observed. This is also compatible with the fact that GLUT2 is a high Km (15–20 mM) GLUT allowing the influx of glucose when extracellular concentrations are high. Moreover, the presence of GLUT1/3 inhibitor did not alter glucose consumption in the SCs of Tg-RGN rats, in opposition to the observed in the Wt group (Fig. 2a). Although GLUT2 was previously identified in the SCs^53^, its functional role has been mostly described in the intestine, pancreas, kidney, and liver, those tissues handling the dietary sugars^54–56^, with this study firstly suggesting the involvement of GLUT2 in the glycolytic metabolism of SCs under conditions of RGN overexpression. The likely role of GLUT2 handling glucose uptake in SCs is also supported by the findings showing three-times higher intratesticular glucose concentrations relative to the serum levels in normoglycemic mice^57^. Nevertheless, it is also plausible to consider that the lower glucose consumption by the SCs of Tg-RGN under high glucose availability may be related with hexokinase inhibition, as high glucose concentrations are known to inhibit hexokinase activity^58,59^.

The diminished glucose consumption detected in the SCs of Tg-RGN rats (Fig. 2a) was accompanied by a decrease in the intracellular glucose concentrations (Fig. 2c), and followed previous findings in other glycolytic tissues. Vaz *et al*.^26^. showed that the *in vivo* overexpression of RGN in the liver, one of the body’s reservoirs of glucose, brain, an organ with a great demand for glucose, and prostate, caused tissue glucose concentrations to decrease. Moreover, the lower glucose concentration found in prostatic tissues was accompanied by the augmented expression levels of PFK1^26^, an enzyme that catalyses a rate-limiting step in glycolysis (Fig. 1), which allowed authors to suggest that glucose was being actively metabolized. Indeed, the increased expression of PFK1 has been associated with a high glycolytic activity which, in turn, is related to a greater glucose flux and metabolization in the cell^60^. Our results followed these observations. The expression levels of PFK1 were highly increased in the SCs of Tg-RGN animals (Fig. 4d), contributing to the diminished intracellular glucose levels, and sustaining a high glycolytic flux.

Lactate, the by-product of glycolysis obtained from pyruvate, is the central energy metabolite used by germ cells, being responsible, among other biological processes, for the stimulation of RNA and protein synthesis in spermatids^61^. It is widely recognized that SCs are the main consumers of glucose in the seminiferous epithelium actively producing lactate to fulfil the metabolic needs of germ cells throughout their development^1,9,10^. We have noted that notwithstanding with the diminished glucose consumption, and despite no significant differences were found in the intracellular lactate levels (Fig. 2d), lactate production was augmented in the SCs of Tg-RGN animals (Fig. 2b). The enhanced export of this glycolytic metabolite was sustained by the increased expression levels of MCT4 (Fig. 4f), the membrane transporter required to export the lactate onto the extracellular space. However, the protein expression (Fig. 4g) and the enzymatic activity (Fig. 5) of LDH were significantly decreased in the SCs of Tg-RGN rats. This led us to hypothesize that other alternative substrates might be getting used for the production of lactate, which is corroborated by the increased lactate production detected both in Wt and Tg-RGN rats SCs (Fig. 2b) when glucose availability was reduced. A recent study has shown that lactate production by SCs is centrally regulated by the known master regulators of glycolytic metabolism, the hypoxia-inducible factors (HIFs)^62^, which raises the curiosity whether these factors can being stimulated by the low glucose concentrations and driving lactate production. Indeed, both hyperglycaemia and low glucose concentrations have been implicated in the regulation of HIF1α expression and stability^63,64^.

It has been shown that in order to maintain high rates of metabolic activity SCs can use amino acids as energy sources, which includes alanine and glutamine but not glycine^65^. The oxidation of glycine by SCs has been considered non-significant for energy purposes^65^. Alanine is a substrate placed at the crossroad of glucose and amino acids metabolism by the activity of ALT, the enzyme that catalyses the reversible reaction of alanine conversion into pyruvate (Fig. 1). A pronounced increase in ALT’s expression was found in the SCs of Tg-RGN animals (Fig. 4e), suggesting that a significant part of the pyruvate produced by these cells may likely have its origin in the conversion of alanine by ALT and not in glycolysis.

Although the contribution of ALT for the production of pyruvate by SCs has been a matter that lacks directed studies, it has been shown that these cells can maintain lactate production even in the total absence of glucose^51^. This further supports the idea that the enhanced lactate production in the SCs of Tg-RGN rats may be driven by the augmented pyruvate production in consequence of increased ALT expression. Further studies would be needed to ascertain the possibility of lipids metabolism being contributing to the modulation of TCA cycle by acetate and acetyl-CoA and, thus, altering pyruvate/lactate availability. This is quite relevant since it is known that the SCs can uptake fatty acids through the fatty acid transporter CD36 (FAT/CD36)^66^ and use the apoptotic germ cells and residual bodies as energy sources by their degradation to form lipids that are conducted to β-oxidation to produce ATP^67^.

It is widely accepted that is the activity of SCs that mostly produces and establishes the composition of the SeT fluid^1,38^. Interestingly, the content of glucose in the SeT fluid of Tg-RGN rats mirrored the SCs behaviour. A higher concentration of glucose was found in the SeT fluid of Tg-RGN rats (Fig. 3a) in accordance with the diminished glucose consumption observed in the SCs (Fig. 2a). Moreover, measurement of glucose concentration in the culture medium of SeT tubules maintained *ex vivo* showed that glucose consumption was lower in the Tg-RGN (Fig. 3c), which is compatible with the diminished glucose consumption observed in SCs cultures alone and with the reduced content of this metabolite in the SeT fluid.

Concerning the energetic substrate lactate, despite the higher production found in the SCs of Tg-RGN (Fig. 2b), the concentration of this metabolite in the SeT fluid was diminished (Fig. 3b). Together with the decreased lactate production observed in the *ex vivo* cultures of Tg-RGN (Fig. 3d), this strongly supports that the lactate produced is being metabolized by the germ cells.

Besides being an energy substrate, it has been indicated that lactate exerts anti-apoptotic effects on germ cells^5,8^, being also shown that the testicular infusion of lactate into adult cryptorchidic rat testis improves the spermatogenic process^68^. Herein, the differential lactate handling by the SCs and SeT of Tg-RGN animals in culture was followed by the diminished apoptosis in the seminiferous epithelium (Fig. 3e). These findings are further supported by a previous study conducted in the whole testis showing that Tg-RGN rats present lower apoptotic rates in consequence of the diminished activity of the apoptosis-effector caspase-3^34^. Also, it has been shown that RGN suppresses thapsigargin- and actinomycin D-induced apoptosis in SeT by modulating the expression and activity of key apoptotic and antiapoptotic factors^25^. Therefore, it is liable to speculate that the RGN beneficial effects in the testis of Tg-RGN rats suppressing apoptosis of germ cells would be associated with the augmented levels of lactate made available for the germ cells in consequence of the enhanced production of SCs, as was demonstrated herein. Moreover, the results obtained are in line with previous findings from our research group also describing the higher sperm viability in the Tg-RGN animals^50^.

Considering glutamine, Grootegoed *et al*.^15^ have demonstrated that the single oxidation of this amino acid yields most of the energy that SCs require. The process of glutaminolysis firstly depends on glutamine entering into the cell, a task that is achieved by the glutamine transporter ASCT2 (Fig. 1)^14^. The SCs of Tg-RGN animals have shown increased expression of the ASCT2 (Fig. 6b), which was very consistent with the higher glutamine consumption (Fig. 6a) observed. Aside supporting the production of anti-oxidant molecules (NADPH and glutathione), glutaminolysis is a mitochondrial pathway (Fig. 1) that involves the initial deamination of glutamine by GLS^69^. Our results have shown that also the expression levels of GLS were increased in the SCs of Tg-RGN animals (Fig. 6c), suggesting a high rate of glutamine-oxidation.

The molecular regulation of glutamine metabolism in testicular cells is poorly known, but in cancer cells, the oncogenic transcription factor c-myc, has been indicated as a central player in the control of glutaminolysis being responsible for the increased expression and activity of ASCT2 and GLS^47,70,71^. The results obtained herein showed that the alterations in glutamine metabolism in the SCs of Tg-RGN were underpinned by the increased expression of c-myc (Fig. 7), which suggests that the RGN actions in the control of metabolism may be indirect. RGN overexpression also has been shown to modulate c-myc expression in hepatoma cells^48^, but the relationship between c-myc and RGN needs further characterization in future work. In sum, glutamine consumption and metabolization were increased in the SCs of Tg-RGN animals, which would be of paramount importance considering the diminished uptake of glucose.

The specific role of glutamine substrate to SCs’ metabolism remains unknown, but it cannot be excluded from the discussion that it can contribute to the final pool of pyruvate/lactate. In both astrocytes^72^ and enterocytes^73^, glutamine utilization to produce lactate via the oxidative pathways of glutamate degradation has been described. In the case of SCs this ability requires further investigation, but it has been shown that glutamine prevents the incorporation of alanine into proteins^65^, a quite relevant issue given the fact that alanine can be converted to pyruvate. Therefore, indirectly, glutamine may be increasing pyruvate by augmenting the intracellular levels of alanine, which in the scenario of SCs of TG-RGN rats could be potentiated by the increased expression of ALT.

The mitochondrial degradation of glutamine’s metabolite, glutamate is initiated by its entry into the TCA cycle and its conversation to α-ketoglutarate. This degradation is a partial process because glutamate cannot be fully degraded by the TCA cycle since this is a catalytic process in which two carbons enter as acetyl-CoA and two carbons are released as CO_2_. A four-carbon molecule must leave the TCA cycle and be converted to pyruvate, which can have several possible fates: re-enter the TCA cycle for complete oxidative degradation of glutamate or be converted to lactate^74^.

The existence of this putative relationship between glutamine metabolism and glycolysis, together with the augmented expression of ALT, may explain the augmented lactate production in the SCs of Tg-RGN rats when the protein expression and enzymatic activity of LDH were diminished. The great metabolic plasticity of SCs has also been shown by other routes and different experimental conditions, namely, under glucose or insulin deprivation and exposure to endocrine disrupting chemicals^51,75,76^, which indicates that the regulation of SCs metabolism is a complex and intricate process that needs to be better understood.

In conclusion, the present findings established RGN as an important regulator of the SCs’ glucose and glutamine metabolism. Despite consuming less glucose, the SCs of Tg-RGN animals display an adaptation of metabolism that maintains high rates of lactate production and exportation. The lower glucose uptake seems to be compensated by the alanine and glutamine metabolism that can drive the generation of pyruvate fuelling the production of lactate. Further work to deeply characterize alanine and glutamine metabolism in SCs is compulsory. Overall, these discoveries extended the array of RGN roles supporting a successful spermatogenesis and highlighted for the plasticity of SCs metabolism, which could be of uttermost importance in the context of male infertility.

## Methods

### Animals and tissue collection

Three-month-old Wt and Tg-RGN Sprague Dawley rats (*Rattus norvegicus*) (Charles River, Barcelona, Spain and Japan SLC, Hamamatsu, Japan, respectively) were maintained with food and water *ad libitum* in a constant room temperature (20 ± 2 °C) on a 12-hour cycle of artificial lighting. All experiments complied with the US National Institutes of Health guidelines^77^ and the European Union rules for the care and handling of laboratory animals (Directive 2010/63/EU). The protocol for animal tissue collection was approved by the local “FCS-UBI Animal Facilities Committee”. All rats (n = 6 in each group) were euthanized under anesthesia (Clorketam 1000, Vetoquinol, Lure, France) and the testes removed. One testis from each animal was used for fluid collection, and the contralateral testis for SCs isolation and SeT culture.

### SeT fluid collection

After testicular excision, the tunica albuginea was peeled back and the SeT exposed were washed in HBSS to remove the interstitial fluid. The SeT fluid was obtained as previously described^32^. In brief, the tubules were extruded through the hub of a 3 mL syringe into a tube, and after a 6000 *g* centrifugation at 4 °C for 15 min, the SeT fluid (supernatant) was collected. SeT fluid was immediately frozen in liquid nitrogen and stored at −80 °C for metabolite analysis.

### Primary SCs culture

Sertoli cells were isolated using an adaptation of the enzymatic procedure described by Aly *et al*. (2010)^32^. Briefly, extruded SeT were incubated in a collagenase solution (0,5 mg/mL in 1XHBSS pH 7.4) at 34 °C for 10–15 min under shaking (80 oscillation/min) and allowed to settle. The tubule fragments were then washed in HBSS and incubated in a trypsin solution (0,5 mg/mL in 1XHBSS pH 7.4) at 37 °C for 5–10 min with gentle shaking. The SCs suspension was collected by centrifugation (250–300 *g* for 3–4 min), washed in HBSS and resuspended in SCs culture medium (DMEM:F-12 (Sigma-Aldrich, St. Louis, Missouri, USA)) supplemented with 50 IU/mL penicillin, 50 mg/mL streptomycin sulfate, 0.5 mg/mL Fungizone, 50 µg/mL gentamicin, and 10% (v/v) heat-inactivated FBS (Biochrom, Berlin, Germany)). The cell suspension was forced through a syringe and plated in culture flasks (Cell+; Sarstedt, Nümbrecht, Germany). The cultures were incubated at 37 °C in an atmosphere of 5% CO_2_ until a 90–95% confluence was achieved. Culture medium was then replaced by serum-free medium supplemented with ITS (Sigma-Aldrich) and the SCs were cultured for 24 hours in standard DMEM:F-12 medium, high (17 mM) glucose concentration, in the presence or absence of a GLUTs inhibitor (400035, Calbiochem, Darmstadt, Germany). SCs from Wt and Tg-RGN animals were also cultured in low (8 mM) glucose concentration to confirm if low glucose availability change the consumption pattern.

### *Ex vivo* culture of rat SeT

Tunica albuginea was cut and peeled back to expose tubules. Ten fragments of SeT were placed in culture plate (Nunclon D 12 well multidishes; Nunc, Roskilde, Denmark) wells containing 2 ml of pre-warmed DMEM: F-12 culture medium (Sigma-Aldrich) supplemented with 20 mg/L gentamicin sulfate, 0.1 mM 3-isobutyl-1-methylxanthine, and 1 µg/L BSA. SeT fragments of 1 cm were used to guarantee the morphometric homogeneity of testicular cell types. The SeT from Wt and Tg-RGN rats were incubated for 48 hours at 33 °C in an atmosphere of 5% CO_2_. At the end of the experiment, culture medium and SeT were recovered, snap–frozen in liquid nitrogen and stored at −80 °C.

### Quantification of glucose, lactate and glutamine

The concentration of glucose and lactate in the SeT fluid and culture medium of SCs and SeT of Tg-RGN and Wt rats was determined through spectrophotometric assays using commercial kits (Spinreact, Girona, Spain) as previously described^26^. Calculations were performed to determine glucose consumption and lactate production by SCs and SeT of both groups over the entire culture period. Specifically, the glucose concentration (mg/dL) in the culture medium was determined, converted to mmol/L, and the total glucose content (mmol) at each experimental time-point was obtained based on sample volume. In the end, glucose consumption was calculated in comparison with the glucose content at 0 hours and normalized for the total cell number in each group, whenever applicable.

Polar and non-polar metabolites were extracted from SCs cultured for 24 hours by a methanol/chloroform/water extraction. Briefly, cells were quenched in liquid nitrogen followed by the addition of 1 mL of cold methanol and 500 µl of chloroform. After defrosting on ice, samples were vortexed for 60 s and sonicated. After the addition of 500 µl of chloroform and ice-cold water, samples were vortexed and centrifuged at 5000 *g* for 15 min at 4 °C. The upper layer (water-soluble metabolites) was collected for quantification of glucose and lactate concentrations. All measurements complied with manufacturer’s instructions and were normalized for the total number of cells in each experimental condition.

The quantification of glutamine in the culture medium of SCs was determined using a commercial kit (NZYtech, Lisbon, Portugal) according to the manufacturers’ instructions. In brief, the concentration of glutamine was determined by measuring the amount of NADP^+^ formed through the combined action of GLS and glutamate dehydrogenase. The amount of NADP^+^ formed, measured at 340 nm, is stoichiometric to the amount of L-glutamine and ammonia in the sample’s volume. The amount of L-glutamine per sample was normalized for the total number of cells in each experimental condition.

### Extraction and quantification of total protein

Total proteins were isolated from SCs and SeT using RIPA buffer (150 mM NaCl, 1% Nonidet-P40 substitute, 0.5% Na-deoxycholate, 0.1% SDS, 50 mM Tris pH 8 and 1 mM EDTA) supplemented with protease inhibitors cocktail (Sigma-Aldrich). The cell lysates were homogenized, centrifuged at 14000 *g*, 20 min, 4 °C and the supernatant containing the protein was collected. Afterwards, protein concentration was determined by the Bradford assay (Bio-Rad, Hercules, CA, USA).

### Caspase-3-like colorimetric activity assay

Caspase-3 activity assay was assessed by determining the cleavage of a colorimetric substrate. Briefly, 25 µg of total protein extracted from SeT were incubated with an appropriate volume of reaction buffer (20 mM HEPES pH 7.4; 2 mM EDTA; 0.1% 3-((3-cholamidopropyl) dimethylammonio)-1-propanesulfonate; 5 mM dithiothreitol) and 200 µM of caspase-3 substrate (Ac-DEVD-p-nitroaniline, Ac-DEVD-pNA) for 2 h at 37 ° C. Upon caspase cleavage of Ac-DEDV-pNA, pNA is released producing a yellow colour, which is measured spectrophotometrically at 405 nm. The activity of caspase-3 is directly proportional to the amount of generated product that was calculated by extrapolation from a standard curve of free pNA. Results were expressed as nM pNA/µg protein.

### Western Blot (WB)

Proteins (50 μg of each protein extract) were resolved in a 12% gel by SDS-PAGE and transferred to a PVDF membrane (Bio-Rad). Membranes were incubated overnight at 4 °C with rabbit anti-GLUT1 (1:500, CBL242, Millipore, MA, USA), rabbit anti-GLUT2 (1:500, sc-9117, Santa Cruz Biotechnology, Dallas, TX, USA), rabbit anti-GLUT3 (1:1000, sc-30107, Santa Cruz Biotechnology), rabbit anti- PFK1 (1:500, sc-67028, Santa Cruz Biotechnology), rabbit anti-MCT4 (1:1000, sc-50329, Santa Cruz Biotechnology), rabbit anti-LDHA (1:10000, Ab52488, Abcam, Cambridge, MA, USA), mouse anti-ALT (1:200, sc-374501, Santa Cruz Biotechnology), rabbit anti-GLS (1:1000, ab93434, Abcam) and rabbit anti-ASCT2 (1:1000, V501, Cell signalling technology, Danvers, MA, USA) primary antibodies. A mouse anti-glyceraldehyde 3-phosphate dehydrogenase (GAPDH) (1:10000, AB2302, Millipore, Darmstadt, Germany) antibody was used for protein loading control in all WB analyses. Goat anti-rabbit IgG-HRP (1:5000, NIF1317; Santa Cruz Biotechnology) or goat anti-mouse IgG + IgM-HRP (1:5000, Santa Cruz Biotechnology) were used as secondary antibodies. The membranes were incubated with ECL substrate (Bio-Rad) and immune-reactive proteins were scanned with the ChemiDoc™ MP Imaging System (Bio-Rad). The density of the bands was obtained according to standard methods using the Image Lab 5.1 software (Bio‐Rad) and normalized by division with the respective GAPDH band density. Results are presented as fold-variation relatively to the control Wt group. For some antibodies, besides the specific band with the predicted molecular weight (Supplementary Fig. 1) other bands were detected. However, in all cases, the specificity of the immunodetection was tested by using tissue positive and negative controls, different sample preparation approaches, and antibody dilutions and incubation times.

### LDH enzymatic activity

The enzymatic activity of LDH in SCs was determined using a commercial kit (Spinreact) according to the manufacturers’ instructions. Lactate dehydrogenase (LDH) catalyses the reduction of pyruvate by NADH and the rate of decrease in concentration of NADPH, measured photometrically, is proportional to the catalytic concentration of LDH present in the sample. The enzymatic activity was calculated by measuring the variation on the absorbance (340 nm) of samples. The method was calibrated using an LDH Positive Control included in the kit. The activities achieved were calculated as U/μg.

### Real-time PCR

Total RNA was isolated from rat SCs using TRI reagent (Sigma-Aldrich) according to the manufacturer’s instructions, and its quantity and integrity were determined by the A260/A280 ratio (NanoPhotometer, Implen, Munich, Germany) and agarose gel electrophoresis. cDNA was synthesized from 1 µg of each RNA sample in a final volume of 20 µl using the First Strand cDNA Synthesis Kit (NZYTech, Lisboa, Portugal) following the protocol supplied by the manufacturer. Synthesized cDNA was stored at −20 °C until further use.

c-Myc expression was analyzed by qPCR using β-actin and GAPDH as internal reference genes. The c-myc, β-actin and GAPDH specific primers sets were (1) sense: AAGAACAAGATG ATG AGGAAG; antisense: GTGCTGGTGAGTAGAGAC; (2) sense: ATGGTGGGTATGGGTCAG; antisense: CAATGCCGTGTTCAATGG; and (3) sense: GTTCAACGGCACAGTCAAG; antisense: CTCAGCACCAGCATCACC, respectively. Reactions were carried out in the CFX Connect™ Real-Time PCR Detection System (Bio-Rad) and the efficiency of amplification was determined for all primer sets using serial dilutions of cDNA. Primer concentration and annealing temperature were optimized and the specificity of the amplicons was determined by melting curve analysis. Each reaction consisted of MaximaTM SYBR Green/Fluorescein qPCR Master Mix (Bio-Rad), sense and anti-sense primers (300 nM for c-myc and 200 nM for the reference genes), and 1 μL of 1:3 diluted cDNA in a final volume of 20 μl. A no-template control was included for each reaction and all reactions were carried out in triplicate. Normalized expression values of c-myc were calculated according to a published mathematical model proposed by Vandesompele and collaborators^78^.

### Statistical Analysis

The statistical significance of differences between Wt and Tg-RGN experimental groups was evaluated through an unpaired Student’s t-test or one-way ANOVA followed by Tukey’s multiple comparison test when applicable using GraphPad Prism v6.00 (GraphPad Software, San Diego, CA, USA). The differences were considered significant when P < 0.05. Experimental data are shown as mean ± S.E.M (n ≥ 4 for each experimental condition).

## Electronic supplementary material

Supplementary Figure S1
