# Supplementary material for: Glucose and glutamine handling in the Sertoli cells of transgenic rats overexpressing regucalcin: plasticity towards lactate production
Source: Sci Rep. 2018 Jul 9;8:10321. doi: 10.1038/s41598-018-28668-4 (PMC6037673; doi:10.1038/s41598-018-28668-4)
Supplement: Supplementary file 1 — Supplementary Figure S1 [file 41598_2018_28668_MOESM1_ESM.pdf]

Glucose and glutamine handling in the Sertoli cells of transgenic rats overexpressing regucalcin: plasticity towards lactate production

Inês Mateus, Mariana Feijó, Luís M. Espínola, Cátia Vaz, Sara Correia[+] and Sílvia Socorro[+,] *

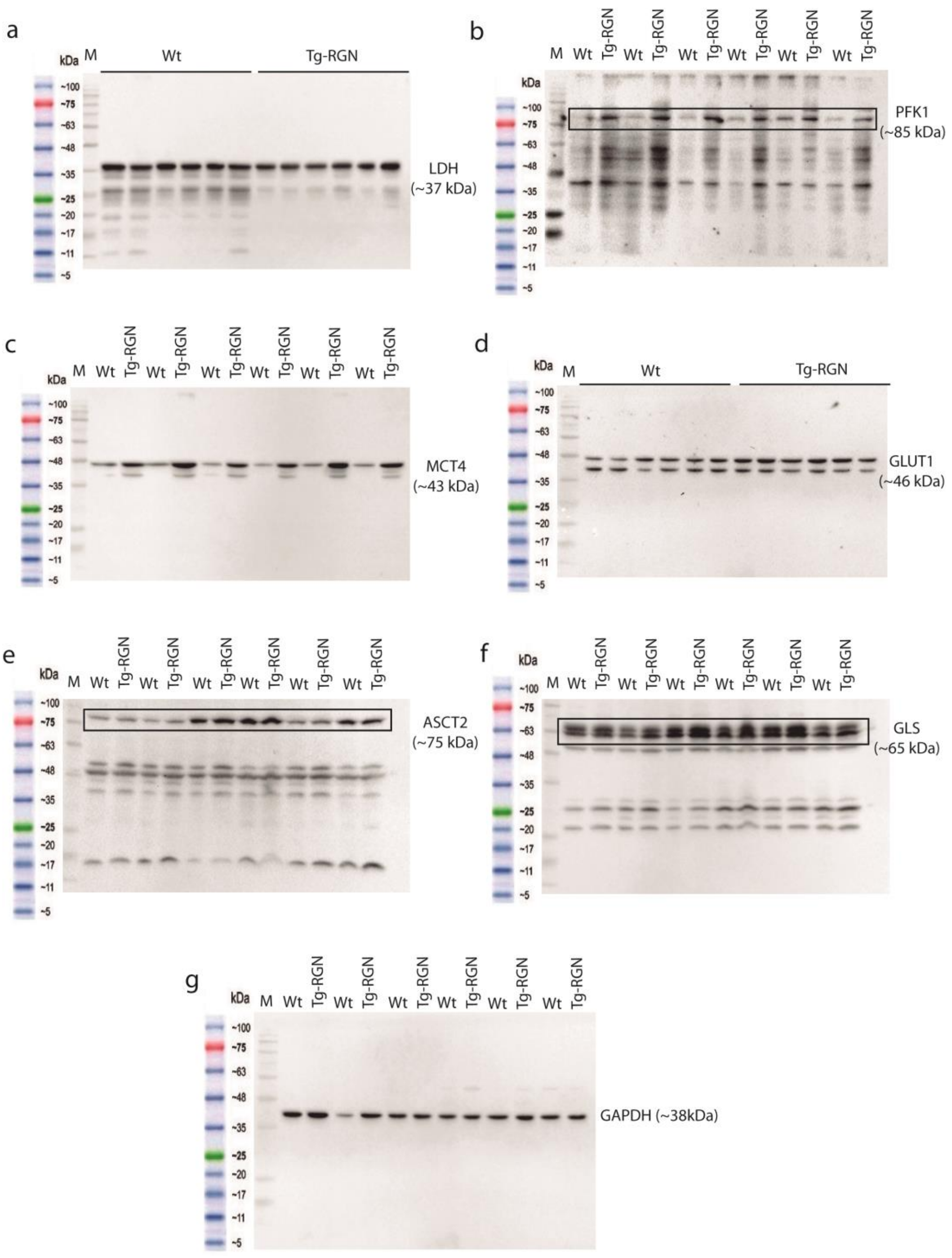

Supplementary figure 1. Full Western blots scans of LDH (**a**), PFK1 (**b**), MCT4 (**c**), GLUT3 (**d**), ASCT2 (**e**), and GLS (**f**) associated with Fig. 4 and Fig. 6. A representative blot of the reference protein for normalization GAPDH is shown (**g**). The protein marker lane (M) and the corresponding molecular weights are shown on the left.
